# Sensorimotor and Sex Influence on Spatial Orientation when Sitting and Standing

**DOI:** 10.1177/00315125251372271

**Published:** 2025-09-03

**Authors:** Josef Rask, Per-Anders Fransson, Rolf Johansson, Måns Magnusson, Fredrik Tjernström

**Affiliations:** 1 Department of Clinical Sciences, 5193 Otorhinolaryngology Head and Neck Surgery, Skåne University Hospital, Lund University, Lund, Sweden; 2 Department of Automatic Control, 5193 Lund University, Lund, Sweden; 3 School of Aviation, 5193 Lund University, Lund, Sweden

**Keywords:** spatial orientation, posture, sex, field dependency, visual dependence, rod-and-frame

## Abstract

When patients experience peripheral vestibular or certain central disorders causing dizziness or vertigo, this is sometimes associated with experiencing also disrupted spatial orientation, which may be accentuated by exposure to a distorted visual environment. This study explores the impact of sensorimotor factors and sex on spatial orientation in sitting and standing positions. In the Rod-and-Frame test, the participant views a rod – with and without a surrounding tilted frame – and is assigned to align the rod vertically or horizontally. A systematic misalignment of the rod due to the tilted frame is considered a visual field dependent behavior. Our objective was to determine whether healthy young adults perform the Rod-and-Frame test equally well when sitting and standing, and to determine the test-retest reliability. Twenty-four participants, 12 males and 12 females (M age = 25.5 years, standard deviation (SD) = 2.8 years) (males M age = 26.3 SD 3.1; females M age = 24.8 SD 2.4), performed identical Rod-and-Frame tests two times – exactly one week between the tests – in a completely dark room while standing and sitting. The participants were assigned to align a luminous rod horizontally and vertically with no frame of reference; with the rod surrounded by a luminous frame tilted 20° clockwise; and 20° counterclockwise. The participants were significantly more field dependent while sitting than while standing during the clockwise frame condition. Females were significantly more field dependent than males in the sitting clockwise and counterclockwise frame conditions. The test-retest evaluation was significantly non-zero for all test conditions and most ICC values were within the “good reliability” range. Thus, during the test conditions with increased sensorimotor information, the participants were less affected by distortive visual information. Additionally, females were more affected by distortive visual information than males while sitting. The Rod-and-Frame test proved robust, providing similar results when repeated.

## Introduction

Postural control is essential for everyday activities like sitting, standing, or walking. Our bodies continuously defy the pull of gravity and keep us from falling despite the postural challenges we are faced with every day. This is possible since several sensory systems in concurrence feed information to our central nervous system (CNS) about both the surroundings and the position and movements of our body. Some of the sensory systems involved are the vestibular organs, proprioception, tactile inputs, and vision (Marieb & Hoehn, 2013). The relative importance of each sensory system is continuously calibrated by the CNS, and changes during our lifespan, depending on function development (Bagust et al., 2013; Ferber-Viart et al., 2007; Jeka et al., 2010; Slaboda et al., 2011). The CNS also continuously produces models about future postural challenges and predicted changes in the environment (Massion, 1992).

When patients experience disorders or lesions causing dizziness or vertigo, the feedback and feedforward systems in postural control may be compromised, forcing the CNS to recalibrate the weighing of the sensory components to maintain spatial orientation and stability control (Marieb & Hoehn, 2013). This can lead to increased dependence on visual cues for balance and thus make these patients more vulnerable to distortive visual inflow (Guerraz et al., 2001), which could cause symptoms like visual vertigo (Bronstein, 1995) or persistent postural-perceptual dizziness (PPPD) (Staab et al., 2017). There are several ways to assess visual dependence. One common way is to evaluate the postural stability under different visual conditions, such as standing with eyes open versus eyes closed, or when viewing a stationary visual stimulus versus a moving stimulus (Kitamura & Matsunaga, 1990). Another commonly used method is to examine the perception of verticality in a confusing visual environment. In daily life there are many objects with a vertical or close-to-vertical orientation, such as trees, houses, and walls. By distorting these visual cues, one may find that the subjective perception of spatial orientation differs, for instance when asking patients to put a rod vertical or horizontal in a room that is perceived to be tilted. Visual dependence in this context is often referred to as field dependence and was first described by Witkin & Asch, 1948 (Witkin & Asch, 1948).

Visual field dependence can also, as in this study, be assessed by a method called the Rod-and-Frame test (Witkin & Asch, 1948), where all visual cues of horizontal/vertical are eliminated except a controlled visual stimulus. The test can be performed by placing a participant in a completely dark room and presenting a luminous rod, either alone or surrounded by a tilted luminous frame. The participant is thereafter asked to align the rod with the perceived vertical or horizontal direction. If a patient is systematically unable to align a rod, not surrounded by a titled frame, vertically or horizontally within an error of about less than 2.5° (Zwergal et al., 2024) the patient is regarded to suffer from a spatial orientation disorder. Participants who are heavily influenced by the frame are considered visual field dependent while participants who put the rod in the correct orientation despite the tilted frame are considered field independent (Guerraz et al., 2001). The tilt of the frame influences the frame effect. Most studies show the largest effects of introducing a tilted frame when the frame is tilted about 10–20° (Wenderoth & Curthoys, 1974; Zoccolotti et al., 1993). In previous research investigating the effects of alcohol intoxication on visual field dependence, it has been observed that counterclockwise (CCW) errors are more common than clockwise (CW) errors, which possibly can be attributed to handedness or eye-dominance (Hafström et al., 2007).

Visual field dependence changes normally during human life. Children and elderly people tend to be more field dependent than adults of 20–39 years (Ferber-Viart et al., 2007; Jeka et al., 2010; Slaboda et al., 2011). One explanation for this might be that when humans grow up, tactile, proprioceptive, and vestibular information become more reliable and therefore vision becomes less important (Bagust et al., 2013; Ferber-Viart et al., 2007). On the other hand, as humans grow old the function of the vestibular system and proprioception wane and vision once again becomes increasingly important (Jeka et al., 2010; Slaboda et al., 2011), especially in fall prone elders (Jeka et al., 2010). Visual field dependence can also change due to disease, treatment, or changes in the environment (Dakin & Rosenberg, 2018). For example, after an experience of weightlessness, astronauts show an increase in visual dependence (Young et al., 1986). Vestibular illness may cause increased visual dependence, though more commonly in patients who suffer from persisting symptoms. For example, patients who had persisting symptoms six months after vestibular neuritis were commonly visually dependent, whereas patients who recovered their vestibular function were not (Cousins et al., 2014). Furthermore, in patients with benign paroxysmal positional vertigo (BPPV), chronic disease seems to cause an increase in visual dependence, but a single attack does not (Agarwal et al., 2012).

A potential factor of relevance is whether performance during Rod-and-Frame tests are dependent on somatosensory properties. Corbett and Enns has shown that participants standing on a tilted surface have a more robust frame effect compared to participants sitting on a tilted surface with the head against a headrest (Corbett & Enns, 2006). Rod-and-Frame tests are performed standing and sitting interchangeably but comparisons between these two test conditions are scarce. Some studies suggest that Rod-and-Frame tests work equally well sitting and standing (Gosselin & Fagan, 2014; Luyat et al., 1997). A smaller frame effect was found only when participants were placed in a supine position (Luyat et al., 1997).

Another potential factor of relevance is whether there might be differences between male and female participants when performing spatial orientation precision tasks during various test conditions. Differences in spatial ability between male and female participants have been found in multiple studies with respect to both the Rod-and-Frame test and many other tests of spatial perception and spatial visualization ability. A meta-analysis investigating sex differences found a highly significant difference that was stable across age for the Rod-and-Frame test, where females were more affected by the frame than male participants (Voyer et al., 1995). These differences emerged around the age of seven, possibly because of a proper understanding of the test at this age and differences in experience with spatial tasks. There was also a difference in performance on the water level task from the age of nine, in which participants are asked to draw a line where the water level would be in a tilted bottle, i.e., estimate horizontality (Voyer et al., 1995). However, it is largely unknown whether sex differences persist through larger changes in the received multi-sensory information that is relevant for determining spatial orientation, e.g., as will be the case when performing spatial orientation tests either sitting or standing. The reasons for the sex differences in spatial ability have been thoroughly investigated and several explanations have been proposed. One proposed model is that genetic potential for spatial ability and extensive exposure to spatial experiences are both needed to excel in spatial tasks (Casey, 1996). This view is supported by a correlation between performance on the water-level task and a childhood preference for toys and sports demanding spatial ability (Voyer et al., 2000). A global study conducted in 2018 investigated spatial ability in navigation using a video game app. 2.5 million people from all over the world participated and two interesting findings emerged. First, spatial ability in the population was found to correlate with economic wealth of the country; and second, sex inequality of a country was predictive of sex differences in navigation ability, with males in unequal countries outperforming the females (Coutrot et al., 2018).

The primary aim of this paper was to investigate whether sitting or standing, and sex influenced the performance on Rod-and-Frame tests in healthy young adults of both sexes. The secondary aim was to determine the reliability of Rod-and-Frame tests when sitting and standing. Our hypotheses were that the spatial orientation error would be about the same when sitting and standing for both sexes, and that the reliability of the Rod-and-Frame tests would be high during all test conditions.

## Method and Materials

### Ethical Treatment of Human Participants

The study was performed in accordance with the latest Declaration of Helsinki and received ethical approval from the Ethical Review Authority in Sweden (Dnr: 2020–06156). The participants were informed verbally and in writing of the test procedure and informed that they could end their participation in the study at any time without any requirement to provide an explanation. A written consent was obtained.

### Participants

Twenty-four healthy adults with normal or corrected-to-normal vision participated voluntarily after signing an informed consent form; twelve males and twelve females (M age = 25.5 years, standard deviation (SD) = 2.8 years) (males M age = 26.3 SD 3.1; females M age = 24.8 SD 2.4). Exclusion criteria were neurological or psychiatric illness, balance-related problems, and sensorineural hearing loss. All participants were asked to refrain from drinking alcohol 24 hours prior to the tests.

### Procedure

The participants performed Rod-and-Frame tests sitting and standing, in identical order for each individual, at the same time of day with one week between the two tests. However, the test order of the individual Rod-and-Frame tests and whether to perform them sitting or standing first was systematically randomized on group level using a Latin-square design. Males and females were randomized separately so that both sex groups were equally distributed. The test sequence performed each week lasted approximately 40 min including the rest periods. All tests were performed without shoes, both when sitting and standing.

**Figure 1.**
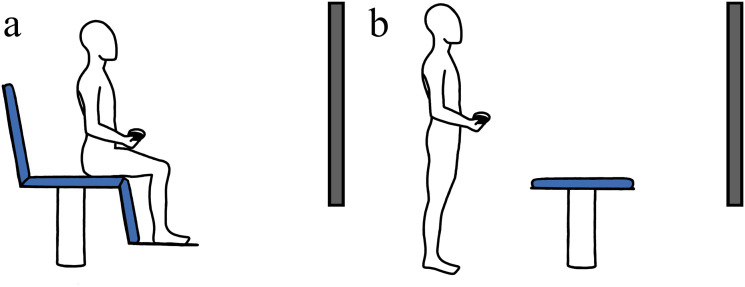
Illustration of posture test conditions. The test participants performed the rod-and-frame tests either sitting in a chair (A) or standing up (B). While seated, the arm rests were elevated, and the backrest was tilted back to 100°, (90° represents completely vertical alignment). The participants were instructed to sit erect without their upper body having contact with the backrest. While standing, the chair was tilted back to 180° (180° represents completely horizontal alignment) and rotated perpendicularly in order not to obstruct the line of view. The participants stood behind the chair without shoes with their feet at a 30° angle and heels 3 cm apart, guided by duct tape on the floor.

While seated, the arm rests were elevated, and the backrest was tilted back to 100°, (90° represents completely vertical alignment) (see Figure 1A). The participants were instructed to sit erect without their upper body having contact with the backrest. All participants had their feet placed on a footrest attached to the chair. While standing, the chair was tilted back to 180° (180° represents completely horizontal alignment) and rotated perpendicularly in order not to obstruct the line of view (see Figure 1B). The participants stood behind the chair without shoes with their feet at a 30° angle and heels 3 cm apart, guided by duct tape on the floor.

**Figure 2.**
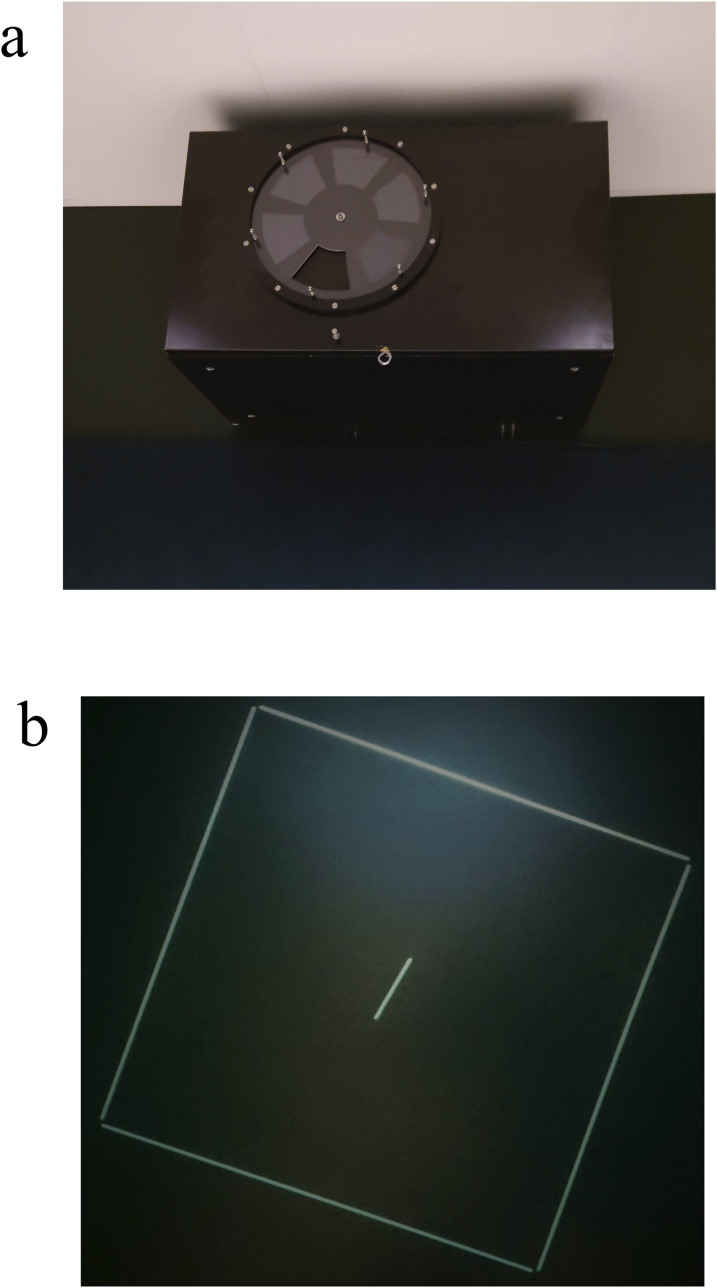
Illustration of the equipment design and the displayed image. (A) The projector was situated inside a custom-made sealed box that prevented any residual light from the projector itself, apart from the image to be displayed, from being emitted into the room where the tests were performed. The image from the projector was displayed through a circular cutout in the box, covered by a neutral density filter that reduced the brightness of the projector light by about 99%. (B) The test participant was instructed to rotate the line in the center to what was perceived as perfectly horizontal or vertical, using a gamepad. In the illustrated case, the frame is rotated 20° clockwise.

A dimly lit, slightly blurry white rod (or both a rod and frame) was projected on a wall 140 cm in front of the participants when sitting and two meters in front of the participants while standing. The projector used for presenting the images was an Infocus IM3118HD (resolution: 1920 × 1080 pixels, brightness: 3000 lumen). The projector was situated inside a custom-made sealed box that prevented any residual light from the projector itself, apart from the image to be displayed, from being emitted into the room where the tests were performed (Figure 2A). The image from the projector was displayed through a circular cutout in the box, covered by a neutral density filter that reduced the brightness of the projector light by about 99%. Limitations in the resolutions of the images displayed by the projector could potentially cause a rod to be displayed choppy, and more so at some angles than others. Care was taken to reduce this effect by using a high-quality HD projector. However, we also found that two other factors markedly smoothed out the rod, so it appeared straight at all angles. These factors were the optical properties of the projector lens used and the use of a neutral density filter that had the main purpose to reduce the brightness of the projector light.

The size and corresponding field of view for the rod was 9 cm × 4 mm (13° sitting, 9° standing), and for the frame it was 59 cm × 57 cm (23° sitting, 16° standing) (Figure 2B). The height of the Rod-and-Frame was not adjusted to eye-level for neither sitting nor standing. The participants were instructed to keep their back and head straight and it was stressed that they should avoid rolling their heads. At the start of the test, the participants were placed in a completely dark room. A rod was then projected at a random angle of ±20–40° from the perfect vertical or horizontal orientation the participants had as task to align the rod with. The rod was displayed together with or without a tilted frame surrounding it depending on test condition. Using the RT and LT-triggers on an Xbox-control, the participants were then instructed to correct the angle of the rod aligned with the participant’s perceived vertical or horizontal orientation. The positioning tasks were repeated four times for each vertical/horizontal orientation test, with all tasks preceded by the rod being projected at a random angle of ± 20–40°. The spatial orientation tests were performed when only a rod was displayed; when a rod was displayed within a frame tilted 20° clockwise, and when a rod was displayed within a frame tilted 20° counterclockwise. It was stressed that the line should be corrected to the gravitational vertical, specifically the participants were told to put the line such that if a marble were dropped at the top of the line, it would fall exactly in the direction of the line. For gravitational horizontal, the participants were told to correct the line to 90° from vertical such that if a marble were to be placed in the middle of the line, it would not roll off on either side.

The orientation of the rod was controlled using three modes. (1) A quick press on a game pad control key, assigned to either clockwise or counterclockwise rotation, made the line rotate a step of 0.05°. Thus, the minimal orientation correction possible was 0.05°. (2) Continuously pressing the control key made the line rotate during the first 4 s around its own center at 1.25°/s. (3) If continuing to press the control key for more than 4 s, the rotation speed then increased to 12.5°/s. When the test participant subjectively perceived the orientation as either perfectly horizontal or vertical, as instructed, the participant pressed a key at the gamepad indicating “accept”. Between each test condition the participants were allowed a short rest and were exposed to light by opening a curtain to hinder night vision adaptation.

From all Rod-and-Frame tests, the rod alignment errors in horizontal and vertical direction were collected for analysis. Alignment errors in clockwise directions (i.e., the rod was rotated too much clockwise) were denoted with a positive sign, and alignment errors in counterclockwise directions (i.e., the rod was rotated too much counterclockwise) were denoted with a negative sign. For spatial orientation tests that included a frame, the frame effect value was calculated by subtracting the value obtained when only a rod was displayed from the value obtained when both a rod and frame were displayed.

### Statistical Analysis

The rod alignment errors were analyzed with both a 4-way and a 2-way repeated measures General Linear model analysis of variance (GLM ANOVA) method. The five dependent variables were: Alignment errors with no frame; Alignment errors with a CW frame; Alignment errors with a CCW frame; Alignment frame effect errors with a CW frame; and Alignment frame effect errors with a CW frame. Values were calculated for horizontal and vertical orientations during all three Rod-and-Frame test conditions, for the first and second test occasion, and for sitting and standing separately. In a first statistical step, and as part of the statistical analysis evaluation of the role of different independent variables, a 4-way repeated measure GLM ANOVA analysis was performed with a model including four independent variables introduced by the test design. The four variables studied were: Rod orientation (Horizontal vs. Vertical) - whether the rod was aligned horizontally or vertically; Test order (Week 1 vs. Week 2) - if the test was conducted in the first or second week; Posture (Sitting vs. Standing) - the participant’s posture during the test; and Sex (Male vs. Female) - the participant’s gender. In the 4-way analyses, the model parameter Rod orientation, Test order and Posture were Within-Subjects factors, and the model parameter Sex was a Between-Groups factor. Using this model revealed that only 2 of the 4 main factors, i.e., Posture and Sex, had a significant influence on the alignment errors recorded. Thus, as a second statistical step, 2-way repeated measures GLM ANOVA analyses were performed with the main factors Posture and Sex on pooled data. Analysis performed on pooled data “allows for better standardization of analytical variables, more robust confounder control, and greater ability to evaluate heterogeneity and effect modification (Omer et al., 2015)”. In the 2-way analyses, the model parameter Posture was a Within-Subjects factor, and the model parameter Sex was a Between-Groups factor. Repeated measures GLM ANOVA analyses were performed after a validation of the appropriateness of using the statistical method, given the properties of the datasets and model residuals, e.g., that the model residuals had normal or close to normal distribution (Knief & Forstmeier, 2021; Midway & White, 2025).

In the Post hoc analyses, Wilcoxon signed rank tests were performed to evaluate the effects of posture and Mann-Whitney U-tests were performed to evaluate the effects of sex. The data in our study fulfils the conditions when it is statistically justifiable to use this approach (Field, 2018; Kirk, 2013; Siegel & Castellan, 1988; Tabachnick et al., 2019). As not all datasets were normally distributed before or after logarithmic transformation, non-parametric statistics were used in the Post hoc statistical evaluation. None of the Wilcoxon signed-rank and Mann-Whitney U-tests evaluations included multiple comparisons, and thus, the significant *p*-value level was set to *p* < .05 also after applying appropriate Bonferroni corrections (Altman, 1991).

An intra-class correlation coefficient (ICC) analysis based on a “2-way mixed” model, an “absolute agreement” analysis, and category “average measures”, was performed to evaluate test-retest reliability. The selection of a “2-way mixed” model refers to that the test equipment will perform the same between repeated testing, but the participants may not identically reproduce their performance between tests. The selection of “absolute agreement” means checking whether the values are identically reproduced and not just correlated. Finally, the option “average measures” stands for that the data from multiple measures was used to calculate the ICC. A higher ICC coefficient value indicates a higher reliability between test-retest values.

Sample size analyses of the parameters revealed an effect size of 0.35 which shows that with the *p*-value set to 0.05 (2-tailed), our study requires 19 subjects to reach a power value of 0.8 for the parameter used. The statistical analyses were performed using SPSS (Version 28, IBM Corp, Armonk, NY, USA) and the power analysis was performed with GPower 3.1.9.7.

## Results

**Figure 3.**
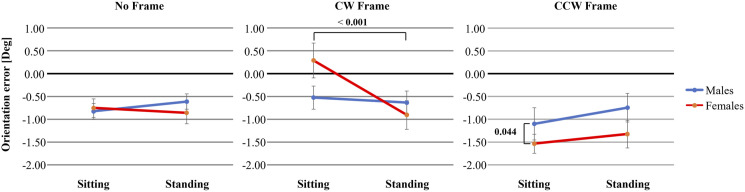
Recorded spatial orientation when tests were performed sitting and standing. The end of the lines illustrates the mean orientation errors in degrees and the whiskers the SEM values. Positive values denote a clockwise rotation. Significant *p*-values are denoted in bold.

**Figure 4.**
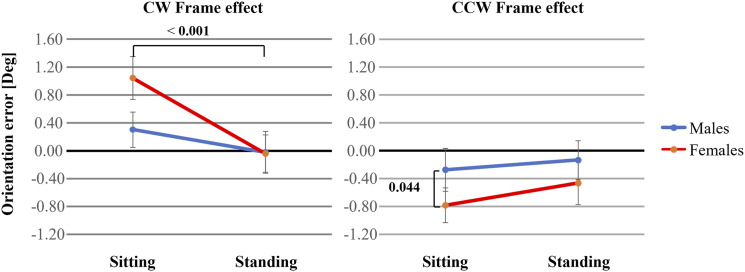
Recorded spatial orientation frame effect when tests were performed sitting and standing. The frame effect is the remaining errors when the errors obtained during the tests when only a rod was displayed are subtracted from the errors obtained when both a rod and a frame were displayed. The end of the lines illustrates the mean additional bias in orientation (in degrees) produced by adding a frame, and the whiskers the SEM values of this bias. Positive values denote a clockwise rotation. Significant *p*-values are denoted in bold.

The basic statistics of the dependent and independent variables relevant to this study are shown in Table 1. From Table 1, it can be observed that the alignment errors from true horizontal and vertical orientation were relatively small in the young healthy participants assessed. The average alignment errors were less than 1.6° from true orientation during all test conditions, both when standing and sitting (Table 1, Figures 3 and 4). Another observation was that almost all alignment errors were negative, i.e., the participants tended to align the rod more CCW than the true orientation.

**Table 1.** Spatial Orientation Errors When Sorted by Variables Sex, Posture, Rod Orientation and Test Order.

Alignment error (degrees)		Dependent variables^a,b^				
Independent variables^c^		No frame	Frame CW^d^	Frame CCW^e^	Frame effect CW^d^	Frame effect CCW^e^
Sex	Male	−0.72 (1.17)	−0.58 (1.76)	−0.92 (2.32)	0.14 (1.92)	−0.27 (2.13)
	Female	−0.81 (1.40)	−0.31 (2.64)	−1.43 (1.45)	0.50 (2.06)	−0.78 (1.74)
Posture	Sitting	−0.79 (1.29)	−0.12 (2.27)	−1.32 (2.02)	0.67 (1.97)	−0.53 (1.95)
	Standing	−0.74 (1.40)	−0.77 (1.98)	−1.04 (2.15)	−0.03 (1.97)	−0.30 (2.03)
Rod orientation	Horizontal	−0.76 (1.27)	−0.30 (2.07)	−1.06 (2.01)	0.45 (2.00)	−0.30 (1.80)
	Vertical	−0.77 (1.43)	−0.59 (2.23)	−1.30 (2.15)	0.18 (1.99)	−0.53 (2.16)
Test order	Week 1	−0.88 (1.27)	−0.43 (2.30)	−1.37 (2.10)	0.45 (2.03)	−0.49 (1.98)
	Week 2	−0.65 (1.41)	−0.46 (2.00)	−0.98 (2.06)	0.19 (1.97)	−0.34 (2.00)

^a^Alignment errors in clockwise directions (i.e., the rod was rotated too much clockwise) is denoted with a positive sign, and alignment errors in counterclockwise directions (i.e., the rod was rotated too much counterclockwise) is denoted with a negative sign.

^b^Presented as mean and standard deviation (SD).

^c^The data collected for each variable subcategory was 384.12 males and 12 females aligned the rod 4 times each in horizontal and vertical direction, 2 times one week apart, for all 5 dependent variables. All participants performed the tests both sitting and standing.

^d^CW = Frame in clockwise orientation.

^e^CCW = Frame in counterclockwise orientation.

### Spatial Orientation Precision

The first statistical step included a 4-way repeated measures GLM ANOVA analysis with main factors Sex, Posture, Rod orientation and Test order. This analysis revealed a significant influence by the main factor Posture in that the alignment errors were significantly smaller when sitting than standing during the CW frame condition (*p* = 0.005) and significantly larger when sitting than standing as CW frame effect (*p* = 0.004) (Table 2). The main factor Sex did not alone have a significant influence on the alignment errors. However, the main factor interaction Posture x Sex revealed that for the CW frame condition, females aligned the rod significantly more CW than males while sitting (*p* = 0.017). The main factors Rod orientation and Test order had no significant effects on spatial orientation precision.

**Table 2.** Spatial Orientation Effects From Posture, Test Order, Rod Orientation and Sex as Determined by a 4-Way Repeated Measures GLM Anova.

Spatial orientation^a,b,c^	Posture	Test order	Rod orientation	Sex	Posture x sex
No frame	0.773 (0.1.0.1, 0.06)	0.188 (1.8, 2.7, 0.29)	0.951 (0.0, 0.0, 0.01)	0.826 (0.0, 0.4, 0.05)	0.386 (0.8, 1.2, 0.19)
Frame CW^d^	0.005 (9.7*, 20.4, 0.66)	0.881 (0.0, 0.0, 0.03)	0.084 (3.3, 4.1, 0.39)	0.729 (0.1, 3.6, 0.07)	0.017 (6.6*, 14.0, 0.55)
Frame CCW^e^	0.231 (1.5, 3.8, 0.26)	0.081 (3.4, 7.2, 0.39)	0.319 (1.0, 2.8, 0.22)	0.497 (0.5, 12.3, 0.15)	0.759 (0.1, 0.2, 0.07)
Frame effect CW^d^	0.004 (10.1*, 23.9, 0.68)	0.190 (1.8, 3.5, 0.29)	0.161 (2.1, 3.8, 0.31)	0.574 (0.3, 6.2, 0.12)	0.102 (2.9, 6.9, 0.36)
Frame effect CCW^e^	0.343 (0.9, 2.5, 0.21)	0.476 (0.5, 1.1, 0.15)	0.405 (0.7, 2.6, 0.18)	0.521 (0.4, 8.4, 0.14)	0.710 (0.1, 0.4, 0.08)

^a^Results are presented for all 4 main factors and for the only significant interaction found. The presented results are *p*-values and (F-values, Sum of squares, Effect size). The notation “< 0.001” means that the *p*-value is smaller than 0.001. Found significant effects are marked in bold text and with an “*“.

^b^All main factors and main factor interactions had a degree of freedom of 1.

^c^For all evaluations, sum of squares values and mean square values were always identical.

^d^CW = Frame in clockwise orientation.

^e^CCW = Frame in counterclockwise orientation.

The second statistical step included a 2-way repeated measures GLM ANOVA analysis with main factors Posture and Sex. The main factor Posture showed that the alignment errors were significantly smaller when sitting than standing during the CW frame condition (*p* < 0.001) and significantly larger when sitting than standing as CW frame effect (*p* < 0.001) (Table 3, Figures 3 and 4). The main factor Sex did not alone have a significant influence on the alignment errors. However, the main factor interaction Posture x Sex revealed that for the CW frame condition, females aligned the rod significantly more CW than males while sitting (*p* < 0.001).

**Table 3.** Spatial Orientation Effects From Posture and Sex as Determined by a 2-Way Repeated Measures GLM ANOVA.

Spatial orientation^a,b,c^	Posture	Sex	Posture x sex
No frame	0.684 (0.2, 0.1, 0.04)	0.720 (0.1, 0.4, 0.04)	0.220 (1.5,1.2, 0.13)
Frame CW^d^	< 0.001 (17.2*, 20.4, 0.43)	0.500 (0.5, 3.6, 0.07)	**<** 0.001 (11.8*, 14.0, 0.35)
Frame CCW^e^	0.072 (3.3, 3.8, 0.19)	0.202 (1.7, 12.3, 0.13)	0.647 (0.2, 0.2, 0.05)
Frame effect CW^d^	< 0.001 (12.6*,\ 23.9, 0.37)	0.302 (1.1, 6.2, 0.11)	0.059 (3.6, 6.9, 0.20)
Frame effect CCW^e^	0.186 (1.8, 2.5, 0.14)	0.257 (1.3, 8.4, 0.12)	0.606 (0.3, 0.4, 0.05)

^a^presented as *p*-values and (F-values, Sum of squares, Effect size). The notation “< 0.001” means that the *p*-value is smaller than 0.001. Found significant effects are marked in bold text and with an “*“.

^b^All main factors and main factor interactions had a degree of freedom of 1.

^c^For all evaluations, sum of squares values and mean square values were always identical.

^d^CW = Frame in clockwise orientation.

^e^CCW = Frame in counterclockwise orientation.

Post hoc analyses of the posture effects showed that females aligned the rod significantly more CW while sitting than standing during the CW frame condition (*p* < 0.001) (Figure 3). Post hoc analyses of the sex effects showed that females aligned the rod significantly more CCW than males while sitting during the CCW frame condition (*p* = 0.044) (Figure 3).

Post hoc analyses of the influence of posture on frame effect data showed that females aligned the rod significantly more CW while sitting than standing during the CW frame condition (*p* < 0.001) (Figure 4). Post hoc analyses of the influence of sex on frame effect data showed that females aligned the rod significantly more CCW than males while sitting during the CCW frame condition (*p* = 0.044) (Figure 4).

### Spatial Orientation Test-Retest Reliability

When investigated with intra-class correlation analyses, the test-retest ICC values were found to be significantly above zero (*p* < 0.001) for all test conditions, both when sitting and standing and both for the spatial orientation precision values and frame effect values (Table 4). Moreover, most ICC values were within the ‘good reliability' range or close to this range.

**Table 4.** Spatial Orientation Test-Retest Reliability.

Spatial orientation^a^	Sitting			Standing		
	Intraclass correlation	95 % confidence intervals	*p*-value	Intraclass correlation	95 % confidence intervals	*p*-value
No frame	0.61	0.31–0.78	< 0.001	0.64	0.36–0.80	< 0.001
Frame CW^b^	0.87	0.77–0.93	< 0.001	0.85	0.74–0.92	< 0.001
Frame CCW^c^	0.87	0.76–0.93	< 0.001	0.82	0.67–0.90	< 0.001
Frame effect CW^b^	0.75	0.55–0.86	< 0.001	0.72	0.50–0.84	< 0.001
Frame effect CCW^c^	0.78	0.60–0.87	< 0.001	0.72	0.51–0.85	< 0.001

^a^The notation “< 0.001” means that the *p*-value for the test against the null hypothesis of zero reliability is smaller than .001. Found significant effects are marked in bold text.

^b^CW = Frame in clockwise orientation.

^c^CCW = Frame in counterclockwise orientation.

## Discussion

Peripheral vestibular lesions and some central disorders causing dizziness or vertigo is sometimes associated with experiencing also disturbed spatial orientation, which may be accentuated by exposure to a distorted visual environment (Bronstein, 1995; Zwergal et al., 2024). However, interoceptive symptoms like spatial orientation malfunctions may not be detected and the size of disorder appropriately assessed when using the standard dizziness and balance disorder tests, e.g., caloric irrigation. It is important that physicians are aware that spatial orientation malfunctions are associated with dizziness and balance disorders and use the appropriate assessment method to detect this. This study explores the test-retest reliability and impact of sensorimotor factors and sex when assessing spatial orientation with the Rod-and-Frame tests.

This study revealed that in general, the rod alignment errors were in CCW direction, even when there was no frame (see Table 1, Figure 3). Consistent with our findings, this has been observed in previous research by Hafström et al., when investigating the effects of alcohol intoxication on visual field dependence (Hafström et al., 2007). Some participants in our study expressed that the CCW frame test was more difficult than the CW frame test. This study also revealed that the frame effect was larger when sitting than standing (see Tables 1–3, Figure 4). One explanation could be that when standing, participants have an increased postural awareness. When similar findings were made by Corbett and Enns comparing sitting to standing, this was attributed mainly to head tilt (Corbett & Enns, 2006). However, since the participants in the current study were instructed to sit freely, without support from a back rest, the effect of the head tilt should be minimal.

Clinical considerations on the Rod-and-Frame test include whether performing the test sitting or standing is better to detect pathology. Since the sitting Rod-and-Frame test gave a larger frame effect in our sample (see Tables 1–3, Figure 4), it could be argued that the tests should be performed sitting. Furthermore, the test-retest reliability presented somewhat higher ICC reliability values when sitting than in standing with respect to both alignment error and frame effect (see Table 4). On the other hand, the mean error was smaller while standing, which could allow for a smaller reference interval and thus potentially better detection of pathology (see Table 1, Figure 4). Another aspect is security, since watching a tilted frame has been shown to increase sway when standing in patients with PPPD (Guerraz et al., 2001). To avoid unnecessary injury, equipment such as a harness should be used to prevent falling. Because of this, a sitting test might be more convenient for the clinic and less stressful for the patient.

In our study, the difference between the sexes was significant for the CW frame for positional errors when sitting, and in post hoc tests also significant for the CCW frame for positional errors and frame effect when sitting (see Table 3, Figures 3 and 4). Thus, the findings suggest that males and females performed the tests more similarly when standing than sitting, and that both sexes performed the tests more accurately when standing than sitting. Hence, sex differences may not persist through larger changes in the received multi-sensory information that is relevant for determining spatial orientation, e.g., as will be the case when performing spatial orientation tests either sitting or standing.

In PPPD, training with optokinetic stimulation has been effective in reducing symptoms of chronic dizziness (Pavlou et al., 2004) and has been shown to decrease field dependence in healthy participants (Pavlou et al., 2011). The high level of test-retest reliability shown in this study advocates that the Rod-and-Frame method is suitable for performing follow up assessments of the treatment effects of PPPD. Most ICC values were within the ‘good reliability' range or close to this range (see Table 4). Prior to clinical application, however, a longitudinal study of this patient group should be conducted to evaluate both the test-retest reliability and learning effect as well as to ascertain how to best account for these factors.

### Study Limitations

A limitation was that the stimulus was not adjusted for the participant’s height. In the data analysis, however, no consistent influence of height was found. Difference in height could possibly incur a more forward head tilt in tall participants when standing, even though this difference in tilt should be small considering the distance to the stimulus. One study has shown that pitch of the environment does not affect the ability to adjust a rod to vertical and does not have an additive effect when the environment is tilted (Nelson & Prinzmetal, 2003), while other studies have found an increase in error when adding a tilt in pitch to the Rod-and-Frame test (Luyat et al., 1997).

Another limitation might potentially be the gap selected between the rod and the frame. A large gap between the rod and a surrounding frame could make the frame effect smaller (Coren & Hoy, 1986) as well as increase the frequency of placement error in the direction opposite to the tilt of the frame (Zoccolotti et al., 1993). The frame effect observed in this study was indeed small: the mean frame effect was less than 2° at most. That said, the sensitivity and effectiveness of the physical properties of the Rod-and-Frame tests used in this study has previously been validated in other research studies involving various clinical populations (Einarsson et al., 2018; Hafström et al., 2004, 2007; Tjernström et al., 2019). Moreover, the intraclass correlation coefficient (ICC) analyses made in this study evidenced a robust performance, in terms of that the tests provided repeatedly the same results also when executed one week apart.

One of the main objectives for the study was to evaluate the reliability of the assessments made when using the test procedures and equipment described in the paper. The randomized protocol means equally often across the study material the correct horizontal and vertical orientation was initially approached from either counterclockwise direction or from clockwise direction. Our ICC evaluation evidenced that when using this approach, the test-retest ICC values were found to be significantly above zero (*p* < 0.001) for all test conditions. A study limitation is that we cannot comment on whether a one-directional approach might have produced better or different results. However, the findings advocate that the test procedure is robust enough to produce reliable results with a randomized dual-directional approach.

Whether the head and body alignment can affect spatial orientation is an intriguing but also complex topic. As is well-known from clinical practice, an important factor is what the CNS perceives as horizontal and vertical using the integrated information from multiple sensory systems. For example, in patients with vestibular disorders, the head can be perfectly aligned, but the patient may still perceive a spatial orientation offset. Similarly, a head misalignment may not produce a distorted spatial orientation because CNS may compensate for this through sensory information. The research about this topic is extensive and we regard that it is beyond the scope of this paper to study whether any changes in the spatial orientation perception were imposed by sitting and standing. However, as a precaution, special care was taken to ensure that the test design did not influence the level of sensory information from the head and neck region, e.g., by not using head rests when sitting. Thus, if there were to be a head misalignment, this condition should be unchanged between sitting and standing. Of note, this study was performed on young participants and none of them displayed visible head or body misalignment.

Finally, the statistical analyses had to address two issues. One statistical issue was that while the scientifical merit and importance of revealing both significant and non-significant variables might be regarded obvious, especially when the research concerns clinical issues, this approach might have effects on the statistical findings made, potentially causing Type II errors. As displayed in Table 2 and Table 3, both the 4-way GLM ANOVA and the 2-way GLM ANOVA found that the variables Posture and Sex had a significant effect on the recorded data. However, the statistical probability for that these observations were correct was rated increased from a 2-star level (p < 0.01) for a 4-way ANOVA, to a 3-star level (p < 0.001) for a 2-way ANOVA. This is likely an effect of when a 4-way GLM ANOVA analyzes the effects of more variables based on the same material, the model estimated will be affected by that the smaller datasets used will have larger estimate variances. Our approach to address this issue was to ensure that the base material used was sufficiently large to allow a reliable 4-way GLM ANOVA analysis and then to perform a more detailed analysis of the variables found significant by using a 2-way GLM ANOVA model.

Another statistical issue was that not all datasets were normally distributed before or after logarithmic transformation. Given the properties of the datasets and model residuals—*i.e*., that the model residuals had normal or close to normal distribution (Knief & Forstmeier, 2021; Midway & White, 2025) —and after a validation of the appropriateness of using GLM ANOVA, repeated GLM ANOVA analyses were performed. In the Post hoc analyses, the non-parametric tests of Wilcoxon signed rank tests and Mann-Whitney U-tests were used after ensuring that the data fulfilled the conditions when it was statistically justifiable to use this approach (Field, 2018; Kirk, 2013; Siegel & Castellan, 1988; Tabachnick et al., 2019).

## Conclusion

When patients experience peripheral vestibular or certain central disorders causing dizziness or vertigo, this is sometimes associated with experiencing also disrupted spatial orientation, which may be accentuated by exposure to a distorted visual environment. While many symptoms of vestibular and central disorders are easy to detect and quantify objectively because they produce a nystagmus or a malfunctioning vestibulo-ocular reflex, diagnosing interoceptive symptoms like a distorted spatial orientation is more challenging, though such symptoms may still have a marked effect on the patient’s quality of life. It is important that physicians are aware of this disorder associated with dizziness and balance disorders and use the appropriate assessment method to detect spatial orientation malfunctions. This study explores the test-retest reliability and impact of sensorimotor factors and sex when assessing spatial orientation with the Rod-and-Frame tests. This study revealed that during the standing test condition with increased sensorimotor information, the participants were less affected by distortive visual information. Additionally, females were more affected than males by the visual distortion while sitting. In general, the Rod-and-Frame spatial orientation tests proved robust in terms of providing repeatedly the same results, also when performed one week apart. This study highlights that some interoceptive symptoms, such as a distorted spatial orientation, can be assessed in detail by using the appropriate test procedures and equipment, and highlights the importance of that more tests of interoceptive symptoms are made available for clinical use by future research.
